# The Effect of Hematocrit on All-Cause Mortality in Geriatric Patients with Hip Fractures: A Prospective Cohort Study

**DOI:** 10.3390/jcm12052010

**Published:** 2023-03-03

**Authors:** Yu-Min Zhang, Kun Li, Wen-Wen Cao, Shao-Hua Chen, Bin-Fei Zhang

**Affiliations:** Department of Joint Surgery, Honghui Hospital, Xi’an Jiaotong University, Beilin District, Xi’an 710054, China

**Keywords:** hip fracture, HCT, all-cause mortality, geriatric medicine

## Abstract

Objective: The present study aimed to evaluate the association between hematocrit (HCT) levels and all-cause mortality in geriatric hip fractures. Methods: Older adult patients with hip fractures were screened between January 2015 and September 2019. The demographic and clinical characteristics of these patients were collected. Linear and nonlinear multivariate Cox regression models were used to identify the association between HCT levels and mortality. Analyses were performed using EmpowerStats and the R software. Results: A total of 2589 patients were included in this study. The mean follow-up period was 38.94 months. Eight hundred and seventy-five (33.8%) patients died due to all-cause mortality. Linear multivariate Cox regression models showed that HCT level was associated with mortality (hazard ratio [HR] = 0.97, 95% confidence interval [CI]: 0.96–0.99, *p* = 0.0002) after adjusting for confounding factors. However, the linear association was unstable and nonlinearity was identified. A HCT level of 28% was the inflection point for prediction. A HCT level of <28% was associated with mortality (HR = 0.91, 95% CI: 0.87–0.95, *p* < 0.0001), whereas a HCT level > 28% was not a risk factor for mortality (HR = 0.99, 95% CI: 0.97–1.01, *p* = 0.3792). We found that the nonlinear association was very stable in the propensity score-matching sensitivity analysis. Conclusions: The HCT level was nonlinearly associated with mortality in geriatric hip fracture patients and could be considered a predictor of mortality in these patients. Registration: ChiCTR2200057323.

## 1. Introduction

Hip fractures are common injuries seen in older adults; they are especially common in women who often suffer from osteoporosis and multiple comorbidities [1]. In the United States, more than 320,000 patients are hospitalized per annum with hip fractures [2]. The global annual number of hip fractures is predicted to increase to 6.5 million fractures by 2050 [3]. A similar upward trend in the incidence of hip fractures has been projected for China, where it is estimated that the number will rise to 1.079 million by 2050 [4]. Although hip fractures comprise only 14% of geriatric fractures, they account for 72% of the total cost of orthopedic fracture care in older adults [5]. Most geriatric hip fractures are caused by falls, and they are associated with an increased mortality. The mean one-year global mortality rate in geriatric hip fracture patients is 22% [6]. Given the aging population and high incidence of hip fractures, the costs of geriatric hip fractures are projected to increase dramatically and consume more medical and health resources [7].

The prognosis of geriatric hip fractures is poor, and only about one-fifth of them return to their preinjury functional status one year after surgery [8]. Multiple factors have been proven to directly affect morbidity and mortality after hip surgery; these include delay in surgery, cancer, coronary heart disease, stroke, pneumonia, urinary tract infection, fluid and electrolyte imbalances, and perioperative anemia [9,10]. In addition, a systematic review indicated that malignancy, nursing home residence, time to surgery > two days, pulmonary disease, diabetes, and cardiovascular disease significantly increased the risk of mortality after hip fracture surgery [11]. Song also reported that frailty can predict adverse outcomes effectively in geriatric hip fracture patients [12].

Hematocrit (HCT), is a test that measures the volume of packed red blood cells relative to whole blood [13]. This test can identify conditions such as anemia or polycythemia, monitor response to treatment [14], and as act as a surrogate marker for factors affecting mortality after hip fracture [15]. 

Anemia, defined by hemoglobin levels in most of these studies, is common in older adults and may increase the risk of death [16]. It was reported that an estimated 40% of geriatric hip fracture patients were anemic on admission, and nearly all of them were anemic postoperatively [17]. Previous studies have identified anemia as a significant prognostic risk factor for patients who undergo surgery for hip fracture [18,19,20,21,22]. Several studies [21,23,24,25] have reported that anemia on admission was associated with short and long-term mortality. A study from Lancet has reported that preoperative low HCT was independently associated with an increased risk of 30-day morbidity and mortality in patients undergoing major surgery [26]. However, as an indicator of anemia, the role of HCT on mortality in hip fracture patients was unclear. Therefore, the specific relationship between HCT levels and the prognosis of patients with hip fractures needs to be further explored.

This study was to assess the influence of HCT level on all-cause mortality in geriatric patients with hip fractures over a long-term follow-up period. we hypothesized that there would be either a linear or a nonlinear association between HCT levels and mortality. In this prospective cohort study, we aimed to identify the role of HCT levels on hip fractures.

## 2. Materials and Methods

### 2.1. Study Design

Older adult patients who had a hip fracture between 1 January 2015, and 30 September 2019, and admitted to the largest trauma center in Northwest China, were enrolled in this study.

This prospective study was approved by the Ethics Committee of Xi’an Honghui Hospital (No. 202201009). All procedures involving human participants were performed in accordance with The Code of Ethics of the World Medical Association (Declaration of Helsinki).

### 2.2. Participants

Data in-hospital, demographic and clinical data of the patients were obtained from their original medical records. The inclusion criteria were as follows: (1) age ≥ 65 years; (2) a radiographic or computed tomography diagnosis of the femoral neck, intertrochanteric, or subtrochanteric fracture; (3) receiving surgical or conservative treatment in a hospital; (4) availability of clinical data during hospitalization; and (5) availability of contact via telephone. Patients who could not be contacted were excluded from this study.

### 2.3. Hospital Treatment

Patients were examined using blood tests on admission, including HCT level. The ultrasonography of cardiac and lower extremity veins to prepare for surgery. Intertrochanteric fractures are often managed with closed/open reduction and internal fixation (ORIF) using a proximal femoral nail anti-rotation device. Femoral neck fractures are often treated with hemiarthroplasty (HA) or total hip arthroplasty (THA) according to the patient’s age. Prophylaxis for deep vein thrombosis was initiated on admission. Upon discharge, the patients were asked to return monthly for the assessment of fracture union or function.

### 2.4. Follow-Up

After discharge, patients’ family members were contacted by telephone between January 2022 and March 2022, to record data on survival, survival time, and activities of daily living. Follow-up was conducted by two medical professionals (Wen-Wen Cao and Shao-Hua Chen) who were trained in follow-up skills for two weeks. Three attempts were made to get in contact with patients. Failure of family members of the patients to maintain channels of communication with the team, resulted in patients being recorded as lost to follow-up.

### 2.5. Endpoint Events

The endpoint event in this study was all-cause mortality after treatment. We defined all-cause mortality as death reported by patients’ family members.

### 2.6. Variables

The variables in our study were: age, sex, occupation, history of allergy, injury mechanism, fracture classification, presence of hypertension, diabetes, coronary heart disease (CHD), arrhythmia, hemorrhagic stroke, ischemic stroke, cancer, multiple injuries, dementia, chronic obstructive pulmonary disease (COPD), hepatitis, gastritis, age-adjusted Charlson comorbidity index (aCCI), time from injury to admission, time from admission to surgery, HCT level, operation time, treatment strategy, blood loss, infusion, transfusion, length of hospital, and follow-up. The dependent variable was all-cause mortality, while the independent variable was the HCT level. The other variables were potential confounding factors.

### 2.7. Statistics Analysis

Continuous variables are reported as mean ± standard deviation (Gaussian distribution) or median (range, skewed distribution). Categorical variables are presented as numbers with proportions. Chi-square (categorical variables), one-way analysis of variance (normal distribution), and Kruskal–Wallis H test (skewed distribution) were used to detect differences between different HCT levels. Univariate and multivariate Cox proportional hazards regression models (three models) were used to test the association between HCT levels and mortality. Model 1 was not adjusted for covariates, Model 2 was minimally adjusted for sociodemographic variables, and Model 3 was fully adjusted for all covariates. To test the robustness of our results, we performed sensitivity analysis. We converted the HCT level into a categorical variable according to the anemia criteria and calculated the *p*-value for the trend to verify the results obtained using HCT level as the continuous variable; we also examined the possibility of nonlinearity. Because Cox proportional hazards regression model-based methods are often suspected to be unable to deal with nonlinear models, the nonlinearity between HCT and mortality was assessed using a Cox proportional hazards regression model with cubic spline functions and smooth curve fitting (the penalized spline method). If nonlinearity was detected, we first calculated the inflection point using a recursive algorithm and subsequently constructed a two-piecewise Cox proportional hazards regression model on both sides of the inflection point. In addition, propensity score matching (PSM) was introduced for comparison between matched groups, and confounding factors were adjusted for in the PSM models.

All analyses were performed using statistical software packages R (http://www.R-project.org, accessed on 1 January 2023, R Foundation) and EmpowerStats (http://www.empowerstats.com, accessed on 1 January 2023, X&Y Solutions Inc., Boston, MA, USA). The hazard ratios (HRs) with 95% confidence intervals (CIs) were calculated. A *p*-value < 0.05 (two-sided) was considered statistically significant.

## 3. Results

### 3.1. Patient Characteristics

From the initial sample of 2887 participants who had hip fractures between January 2015 and September 2019, 2589 met the study criteria and were enrolled in our study. The 1-year mortality was 11.05% (286/2303). The mean follow-up period was 38.94 months. Of these, 875 (33.8%) patients died due to all-cause mortality. The HCT levels were divided into four groups. Table 1 lists the demographic and clinical characteristics of all enrolled patients, including comorbidities, factors associated with injuries, and treatment.

### 3.2. Univariate Analysis of Association between Variables and Mortality

To identify potential confounding factors and the relationship between variables and mortality, we performed univariate analysis. According to the criteria of *p* < 0.1, the following variables were considered in the multivariate Cox regression: age (HR = 1.08; 95% CI: 1.06–1.09); *p* < 0.0001), sex (HR = 0.74; 95% CI: 0.65–0.85); *p* < 0.0001), time to admission (HR = 1.00; 95% CI: 1.00, 1.00; *p* = 0.0531), hypertension (HR = 1.13; 95% CI: 0.99–1.29; *p* = 0.0643), CHD (HR = 1.32; 95% CI: 1.15–1.51; *p* < 0.0001), arrhythmia (HR = 1.32; 95% CI: 1.15–1.51; *p* < 0.0001), ischemic stroke (HR = 1.42; 95% CI: 1.24–1.64; *p* < 0.0001), cancer (HR = 1.77; 95% CI: 1.28–2.44; *p* = 0.0005), dementia (HR = 2.62; 95% CI: 2.03–3.38; *p* < 0.0001), COPD (HR = 1.55; 95% CI: 1.23–1.95; *p* = 0.0002), hepatitis (HR = 1.62; 95% CI: 1.17–2.23; *p* = 0.0033), aCCI (HR = 1.51; 95% CI: 1.43–1.61; *p* < 0.0001), time to operation (HR = 1.03; 95% CI: 1.00–1.05; *p* < 0.0481), treatment strategy (ORIF, HA and THA *p* < 0.0001 compared to conservation, respectively), operation time (HR = 1.00; 95% CI: 1.00, 1.00; *p* = 0.0433), infusion (HR = 1.00; 95% CI: 1.00, 1.00; *p* = 0.4246), and length in hospital (HR = 1.02; 95% CI: 1.01–1.04; *p* = 0.0041).

### 3.3. Multivariate Analysis of Association between HCT and Mortality

We used three models (Table 2) to correlate the HCT levels and mortality. Linear regression was observed when HCT level was a continuous variable. The fully adjusted model showed a decrease in mortality risk of 3% (HR = 0.97, 95% CI: 0.96–0.99, *p* = 0.0002) when HCT level increased by 1% after controlling for confounding factors. When HCT level was used as a categorical variable, we found significant differences in HCT levels among the three models (*p* < 0.05). In addition, the *p*-value for the trend also showed a linear correlation in the three models (*p* < 0.05). 

We found the interval to be abnormal in the subgroup with an HCT level above the third quartile (Table 2). This instability indicates the possibility of a nonlinear correlation.

**Figure 1:**
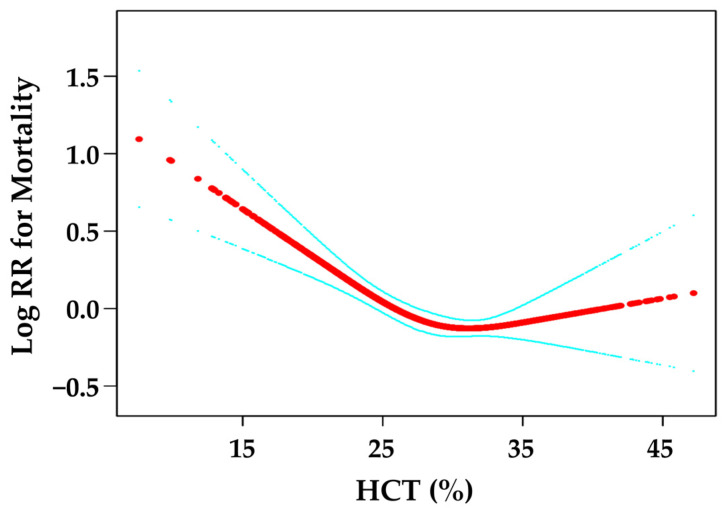
Curve fitting between HCT and mortality. The red line is the fitting curve, and the blue lines are 95% CI.

As shown in Figure 1, there was a curved association between the HCT levels and mortality after adjusting for confounding factors. We compared two fitting models to explain this association (Table 3). Interestingly, we observed an inflection point. A HCT level < 28% was associated with mortality (HR = 0.91, 95% CI: 0.87–0.95, *p* < 0.0001). At a HCT level of >28%, mortality did not change (HR = 0.99, 95% CI: 0.97–1.01, *p* = 0.3792).

### 3.4. Propensity Score Matching (PSM)

To test the robustness of our results, we performed sensitivity analysis using PSM. Overall, 1400 patients (54.07%) were successfully matched. Age (*p* < 0.0001) and aCCI (*p* < 0.0001) did not match between the two groups. In the multivariate Cox regression results under the PSM and PSM-adjusted models, the results were stable, and the inflection point was 29.7% (Table 4).

**Figure 2:**
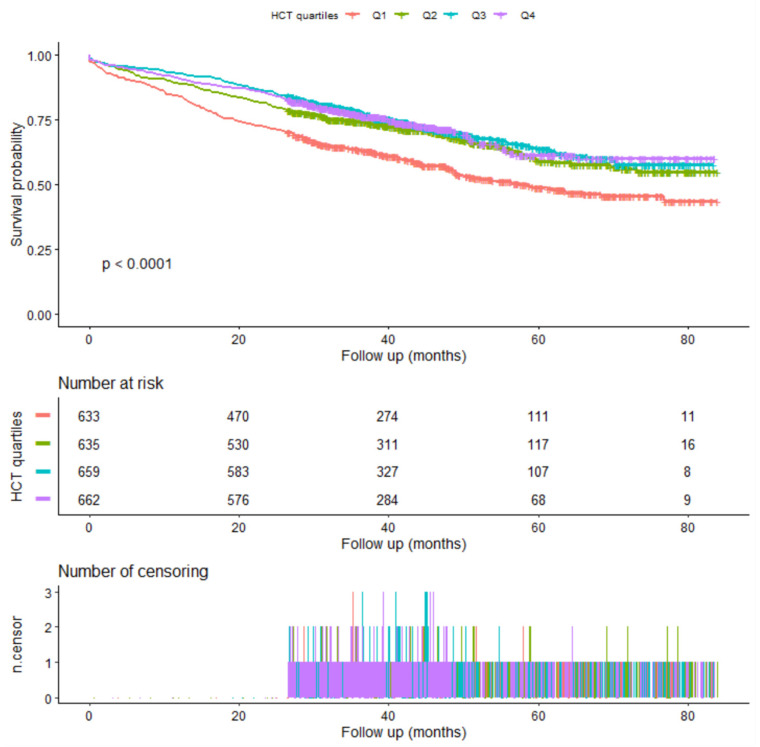
Kaplan-Meier survival.

The Kaplan–Meier survival curve is shown in Figure 2.

## 4. Discussion

A few systematic reviews have indicated many risk factors for mortality in geriatric hip fracture patients [11,12,27,28,29], and anemia was an important prognostic risk factor [18,19,20,21,22]. Even though previous studies have proven the association between preoperative hemoglobin and mortality in hip fracture, the results were an almost linear relationship and did not find nonlinearity. In addition, several high-quality studies assessed the effect of preoperative HCT on mortality in patients undergoing major surgery [26,30,31], but the role of HCT on mortality in hip fracture has not been addressed. Therefore, the specific relationship between HCT levels and the prognosis of patients with hip fractures is needed.

Our study showed a nonlinear association between HCT levels and all-cause mortality in geriatric patients after hip fracture treatment. When the HCT level was <28%, geriatric hip fracture patients with lower HCT levels had greater odds of mortality (HR = 0.91, 95% CI: 0.87–0.95; *p* < 0.0001). This result suggests that a 1% increase in HCT level was associated with a 9% decrease in mortality in geriatric patients with hip fractures. However, when the HCT level was >28%, no association was found between HCT level and mortality in geriatric hip fracture patients (HR = 0.99, 95% CI: 0.97–1.01; *p* = 0.3792). Consequently, the HCT level on admission can be used as a clinical predictor of all-cause mortality in geriatric patients with hip fracture.

There were two primary meanings provided in this paper. On the one hand, the HCT level on admission (<28%) can be used as a clinical predictor of all-cause mortality in geriatric patients with hip fractures. When a patient was on admission, the surgeons should notice that this person was at a high risk of a bad prognosis. On the other hand, we would note the importance of HCT from this study. The surgeons could undertake the intervention study on improving the prognosis by transfusion for HCT < 28% in the future. Specifically, rotational thromboelastometry is a laboratory method that is gaining ground on the evaluation of the hemostatic profile of hip fracture patients [32,33].

A number of previous studies have Investigated the effect of anemia on the prognosis of patients undergoing orthopedic surgery. A single-center retrospective study in Singapore reported that anemia was independently associated with prolonged hospitalization and increased perioperative blood transfusions [34]. Nissenholtz et al. [23] reported that anemia on admission was associated with short and long-term mortality, in addition to the length of stay, amount of blood transfusions, repeated hospitalizations, post-operative complications, poor functioning, and a reduced quality of life. In addition, few studies have examined the effects of anemia on patients with hip fractures. Ryan et al. [21] suggested that geriatric hip fracture patients with preoperative anemia are at an increased risk for morbidity and mortality, especially during the first 30 postoperative days. Similar to the findings of our study, Gruson et al. [24] reported that anemia was associated with a longer length of hospital stay and higher rates of 6-month mortality after surgery for hip fracture. Bolton et al. [25] reported that anemia was a statistically-significant risk factor for perioperative complications. However, most of these studies used hemoglobin levels to define anemia. The specific relationship between HCT levels and prognosis of patients with hip fractures remains unclear. In a systematic review, Sheehan et al. [35] reported frailty was the proposed mechanism for the association between anemia and functional outcome. 

In this study, we found a linear relationship between HCT levels and mortality in geriatric hip fractures; however, we also observed that the relationship was unstable. Therefore, we surmised the possibility of a curvilinear relationship from subgroup analysis and curve fitting. Our study found an inflection point on the curve, which was stabilized by PSM sensitivity analysis. The curvilinear relationship more appropriately explains the association between HCT levels and mortality in geriatric hip fracture patients. Many studies have indicated that HCT levels are associated with organ senescence or complications in older population. In a cohort study of geriatric patients undergoing spinal procedures based on the ACS-NSQIP database, Almeida et al. [36] concluded that patients with lower HCT levels were at a greater risk for requiring transfusion, renal failure, and infectious complications. Gupta et al. [31] found that a HCT level of ≤39% is associated with an increased risk of 30-day mortality and adverse cardiac events in patients aged 65 years or older undergoing elective vascular procedures. Bodewes et al. [37] reported that mortality and major adverse events in patients with chronic limb-threatening ischemia who underwent infrainguinal bypass were inversely associated with preoperative HCT levels.

The patients in our study received blood tests on admission immediately, and the HCT is the first value at admission before treatment. Because of the nature of observational association, we did not give the identified or particular intervention to the patients. However, we gave the transfusion to patients with severe anemia after admission or during the operation in clinical practice. The total volume of the transfusion is 1.84 (U), 1.22 (U), 1.00 (U), and 0.63 (U) in groups Q1–Q4, respectively.

To identify confounders in the study and draw reliable conclusions, we identified factors that affect the HCT level as well as the prognosis of geriatric hip fractures. There were several factors very well known to contribute to mortality in hip fracture population. Age, sex, comorbidities, CHD, arrhythmia, cancer, dementia, time to operation, and treatment strategy were the risk factors for the prognosis of hip fracture which have been reported in previous studies [7,8,10,38,39,40,41,42,43]. The probability of mortality density for a period of 10 years following a hip fracture was 16% for women and 25% for men [44]. A recent systematic review from 81 articles showed that the comorbidities, delay in operation, and type of fractures were important predictors of poor functional outcomes and mortality for patients with hip fractures [28]. In addition, the univariate analysis also found some variables with *p* < 0.1 to be associated ischemic stroke, operation time, and infusion volume. Furthermore, considering factors affecting the HCT level, we included hepatitis, COPD, and cancer [45,46,47,48]. Consequently, we have controlled for the vast majority of confounders.

Our study has a few limitations. First, due to the prospective design of our study, there was an inevitable risk for patients to be loss to follow-up. Patients or their families who could not be contacted initially were called two other times to obtain information regarding patients’ prognosis. Second, the causal relationship between the HCT level and prognosis of hip fractures was not identified in our study and requires further confirmation in future studies. It would be meaningful if future studies could establish a causal relationship between HCT levels and all-cause mortality in geriatric hip fractures. Furthermore, the HCT value was not only influenced by anemia, but also by other factors (hematological disorders or fluid intake). Therefore, generalizations of this conclusion for populations from other regions should be made with caution.

In summary, the HCT level was nonlinearly associated with mortality in geriatric hip fractures and could be considered a predictor of the risk of mortality.

## Figures and Tables

**Table 1:** The Demographic and clinical characteristics (N = 2589).

HCT Quartiles	Q1	Q2	Q3	Q4	*p*-Value	*p*-Value *
N	633	635	659	662		
HCT (%)	26.16 ± 2.94	31.78 ± 1.13	35.47 ± 1.02	40.47 ± 2.80	<0.001	<0.001
Age (year)	81.87 ± 6.47	79.96 ± 6.56	78.98 ± 6.47	77.65 ± 6.97	<0.001	<0.001
Sex					<0.001	-
Male	156 (24.64%)	186 (29.29%)	217 (32.93%)	290 (43.81%)		
Female	477 (75.36%)	449 (70.71%)	442 (67.07%)	372 (56.19%)		
Occupation					0.103	-
Retirement	347 (54.82%)	350 (55.12%)	395 (59.94%)	397 (59.97%)		
Farmer	164 (25.91%)	151 (23.78%)	162 (24.58%)	152 (22.96%)		
Other	122 (19.27%)	134 (21.10%)	102 (15.48%)	113 (17.07%)		
History of allergy	25 (3.95%)	17 (2.68%)	32 (4.86%)	27 (4.08%)	0.241	-
Injury mechanism					0.397	-
Falling	614 (97.00%)	614 (96.69%)	635 (96.36%)	638 (96.37%)		
Accident	12 (1.90%)	17 (2.68%)	22 (3.34%)	17 (2.57%)		
Other	7 (1.11%)	4 (0.63%)	2 (0.30%)	7 (1.06%)		
Fracture classification					<0.001	-
Intertrochanteric fracture	558 (88.15%)	520 (81.89%)	467 (70.86%)	342 (51.66%)		
Femoral neck fracture	53 (8.37%)	95 (14.96%)	176 (26.71%)	311 (46.98%)		
Subtrochanteric fracture	22 (3.48%)	20 (3.15%)	16 (2.43%)	9 (1.36%)		
Hypertension	277 (43.76%)	293 (46.14%)	341 (51.75%)	347 (52.42%)	0.003	-
Diabetes	107 (16.90%)	116 (18.27%)	145 (22.00%)	148 (22.36%)	0.03	-
CHD	335 (52.92%)	352 (55.43%)	336 (50.99%)	354 (53.47%)	0.456	-
Arrhythmia	227 (35.86%)	206 (32.44%)	196 (29.74%)	243 (36.71%)	0.028	-
Hemorrhagic stroke	17 (2.69%)	11 (1.73%)	12 (1.82%)	17 (2.57%)	0.533	-
Ischemic stroke	167 (26.38%)	180 (28.35%)	190 (28.83%)	218 (32.93%)	0.067	-
Cancer	28 (4.42%)	21 (3.31%)	14 (2.12%)	12 (1.81%)	0.02	-
Multiple injuries	64 (10.11%)	48 (7.56%)	43 (6.53%)	26 (3.93%)	<0.001	-
Dementia	37 (5.85%)	22 (3.46%)	23 (3.49%)	21 (3.17%)	0.051	-
COPD	40 (6.32%)	37 (5.83%)	51 (7.74%)	40 (6.04%)	0.493	-
Hepatitis	32 (5.06%)	14 (2.20%)	19 (2.88%)	16 (2.42%)	0.013	-
Gastritis	16 (2.53%)	14 (2.20%)	9 (1.37%)	7 (1.06%)	0.15	-
aCCI	4.43 ± 1.03	4.21 ± 1.05	4.19 ± 1.12	4.05 ± 1.10	<0.001	<0.001
Treatment Strategy					<0.001	-
Conservation	75 (11.85%)	53 (8.35%)	46 (6.98%)	51 (7.70%)		
ORIF	503 (79.46%)	482 (75.91%)	447 (67.83%)	311 (46.98%)		
HA	54 (8.53%)	97 (15.28%)	160 (24.28%)	275 (41.54%)		
THA	1 (0.16%)	3 (0.47%)	6 (0.91%)	25 (3.78%)		
Time to admission (h)	69.30 ± 146.65	93.35 ± 310.53	82.37 ± 252.96	78.56 ± 253.14	0.382	<0.001
Time to operation (d)	4.47 ± 2.64	4.26 ± 2.47	4.16 ± 2.61	4.35 ± 2.58	0.21	0.071
Operation time (mins)	98.18 ± 41.49	91.15 ± 33.78	94.53 ± 40.22	92.67 ± 32.26	0.01	0.072
Blood loss (mL)	271.03 ± 201.70	240.86 ± 152.99	242.65 ± 145.87	229.05 ± 143.63	<0.001	0.375
Infusion (mL)	1488.79 ± 407.67	1511.17 ± 386.41	1587.45 ± 384.04	1642.77 ± 356.39	<0.001	<0.001
Transfusion (U)	1.84 ± 1.35	1.22 ± 1.22	1.00 ± 1.19	0.63 ± 1.02	<0.001	<0.001
Length of hospital (d)	9.17 ± 3.59	8.86 ± 3.77	8.82 ± 3.62	8.86 ± 3.77	0.281	0.031
Follow up (months)	36.96 ± 22.01	40.53 ± 20.81	40.57 ± 18.27	37.70 ± 17.11	<0.001	<0.001
Mortality	288 (45.50%)	212 (33.39%)	192 (29.14%)	183 (27.64%)	<0.001	-

Mean + SD/N(%). *p*-value *: For continuous variables, we used the Kruskal Wallis rank-sum test, and Fisher’s exact probability test for countvariables with a theoretical number < 10. HCT, Hematocrit; CHD, coronary heart disease; COPD, chronic obstructive pulmonary disease; aCCI, age-adjusted Charlson comorbidity index.

**Table 2:** Univariate and multivariate results by cox regression (N = 2589).

Exposure	Non-Adjusted	Minimally-Adjusted Model	Fully-Adjusted Model
HCT	0.96 (0.95, 0.97) < 0.0001	0.97 (0.96, 0.98) < 0.0001	0.97 (0.96, 0.99) 0.0002
HCT quartiles			
Q1	1	1	1
Q2	0.67 (0.56, 0.80) < 0.0001	0.75 (0.63, 0.90) 0.0017	0.78 (0.63, 0.95) 0.0158
Q3	0.59 (0.49, 0.70) < 0.0001	0.68 (0.56, 0.81) < 0.0001	0.72 (0.58, 0.90) 0.0034
Q4	0.60 (0.50, 0.72) < 0.0001	0.71 (0.58, 0.85) 0.0004	0.72 (0.56, 0.91) 0.0062
*p* for trend	<0.0001	<0.0001	0.0036

Data in table: HR (95%CI) *p*-value. Outcome variable: mortality. Exposed variables: HCT. Minimally-adjusted adjust for: age, sex. Fully-adjusted model adjust for: age, sex, time to admission, hypertension, CHD, arrhythmia, ischemic stroke, cancer, dementia, COPD, hepatitis, aCCI, time to operation, treatment strategy, operation time, infusion, and length in hospital. HCT, Hematocrit.

**Table 3:** Nonlinearity of HCT and mortality (N = 2589).

Outcome:	HR (95%CI) *p*-Value
Fitting model by stand linear regression	0.97 (0.96, 0.99) 0.0002
Fitting model by two-piecewise linear regression	
Inflection point	28
<28	0.91 (0.87, 0.95) < 0.0001
>28	0.99 (0.97, 1.01) 0.3792
*p* for log-likelihood ratio test	0.002

Adjusted for age, sex, time to admission, hypertension, CHD, arrhythmia, ischemic stroke, cancer, dementia, COPD, hepatitis, aCCI, time to operation, treatment strategy, operation time, infusion, and length in hospital. HR, hazard ratios.

**Table 4:** Multivariate results by cox regression in PSM model (N = 1400).

	Nonlinearity Model	PSM Model	PSM-Adjust Model
Fitting model by stand linear regression	0.97 (0.96, 0.99) 0.0002	0.99 (0.98, 1.00) 0.1328	0.99 (0.97, 1.00) 0.0381
Fitting model by two-piecewise linear regression			
Inflection point	28	29.7	29.7
<Inflection point	0.91 (0.87, 0.95) < 0.0001	0.95 (0.91, 0.98) 0.0010	0.95 (0.91, 0.98) 0.0007
>Inflection point	0.99 (0.97, 1.01) 0.3792	1.01 (0.99, 1.03) 0.2740	0.95 (0.91, 0.98) 0.0007
*p* for log-likelihood ratio test	0.002	0.005	0.008

Data in table: HR (95% CI) *p*-value. Outcome variable: mortality. Exposed variables: HCT. Adjust variables in PSM-adjusted model: age, aCCI. PSM, Propensity Score Matching.

## Data Availability

The data were provided by Xi’an Honghui Hospital. According to relevant regulations, the data cannot be shared, but could request from correspondence author.

## Abbreviations

aCCI	age-adjusted Charlson comorbidity index
CHD	coronary heart disease
CI	confidence interval
COPD	chronic obstructive pulmonary disease
HCT	hematocrit
HR	Hazard ratio
PSM	propensity score matching
